# Supplementary material for: Reconciling Mining with the Conservation of Cave Biodiversity: A Quantitative Baseline to Help Establish Conservation Priorities
Source: PLoS One. 2016 Dec 20;11(12):e0168348. doi: 10.1371/journal.pone.0168348 (PMC5173368; doi:10.1371/journal.pone.0168348)
Supplement: S1 Dataset — (ZIP) [file pone.0168348.s002.zip › Taxa/Serra Norte/SN_2013/N5S/Lista N5S-35.pdf]

## CAVIDADE N5S-0035

| Classe | Ordem | Fam/Outros | Gên/Outros | Espécie | S-U | U-S | Zon. |
|---|---|---|---|---|---|---|---|
| Arachnida | Acari | (Oribatida) | | sp.3 | X | | E |
| Arachnida | Acari | (Parasitengonina) | | sp.1 | X | | P |
| Arachnida | Acari | (Parasitengonina) | | sp.2 | | X | P |
| Arachnida | Acari | (Parasitengonina) | | sp.5 | | X | P |
| Arachnida | Acari | Argasidae | *Ornithodoros* | sp.1 | X | | P |
| Arachnida | Acari | Ixodidae | *Amblyomma* | sp.1 | X | | E |
| Arachnida | Amblypygi | Phrynidae | *Heterophrynus* | *longicornis* | | 3 | E/P |
| Arachnida | Araneae | Araneidae | | jovem | X | X | E |
| Arachnida | Araneae | Ctenidae | | jovem | 2 | 1 | P |
| Arachnida | Araneae | Ochyroceratidae | | jovem | X | | E |
| Arachnida | Araneae | Pholcidae | *Mesabolivar* | *eberhard* | X | X | P |
| Arachnida | Araneae | Salticidae | | sp.2 | X | | P |
| Arachnida | Araneae | Tetrablemmidae | *Matta* | sp.1 | X | X | P |
| Arachnida | Araneae | Tetragnathidae | | jovem | X | | E |
| Arachnida | Araneae | Theridiidae | | jovem | | X | P |
| Arachnida | Araneae | Theridiosomatidae | *Plato* | sp.1 | X | X | P |
| Arachnida | Opiliones | Sclerosomatidae | | jovem | | X | P |
| Arachnida | Opiliones | Stygnidae | *Paraphareus* | sp.1 | | 2 | P |
| Arachnida | Pseudoscorpiones | Chernetidae | *Spelaeochernes* | sp.1 | X | X | E/P |
| Chilopoda | Scolopendromorpha | Scolopocryptopidae | *Dinocryptops* | *miersii* | 1 | | P |
| Diplopoda | Polydesmida | Fuhrmannodesmidae | | sp.2 | X | | E |
| Diplopoda | Polydesmida | Paradoxosomatidae | | sp.2 | | 1 | E |
| Entognatha | Collembola | Entomobryidae | | sp.1 | X | | E |
| Entognatha | Collembola | Paronellidae | | sp.1 | X | X | E/P |
| Entognatha | Collembola | Paronellidae | | sp.4 | | X | P |
| Entognatha | Collembola | Paronellidae | | sp.6 | X | | E |
| Entognatha | Diplura | Campodeidae | | sp.1 | X | X | E/P |
| Entognatha | Diplura | Projapygidae | | sp.1 | | X | E/P |
| Entognatha | Diplura | Projapygidae | | sp.2 | | X | P |
| Insecta | Blattodea | | | jovem | 2 | | P |
| Insecta | Coleoptera | Elateridae | | jovem | X | | E |
| Insecta | Coleoptera | Scydmaenidae | | sp.1 | X | | E |
| Insecta | Coleoptera | Staphylinidae | Pselaphinae | sp.10 | X | | E |
| Insecta | Diptera | Cecidomyiidae | Cecidomyiinae | sp. | X | | E |
| Insecta | Diptera | Ceratopogonidae | | jovem | X | | E |
| Insecta | Diptera | Conopidae | *Stylogaster* | sp. | X | | P |
| Insecta | Diptera | Drosophilidae | | sp. | X | | E |
| Insecta | Diptera | Psychodidae | *Pintomyia* | *gruta* | | X | E |
| Insecta | Diptera | Psychodidae | *Sciopemyia* | *sordellii* | X | X | P |
| Insecta | Diptera | Sciaridae | *Bradysia* | sp. | X | | E |
| Insecta | Heteroptera | Reduviidae | Reduviinae | jovem | 3 | 1 | P |
| Insecta | Heteroptera | Schizopteridae | Schizopterinae | jovem | X | | P |
| Insecta | Homoptera | Cixiidae | | sp.5 | X | X | E |
| Insecta | Hymenoptera | Formicidae | *Apterostigma* | sp.1 | | X | E |
| Insecta | Hymenoptera | Formicidae | *Cyphomyrmex* | sp.2 | X | | P |
| Insecta | Hymenoptera | Formicidae | *Pachycondyla* | *striata* | X | | E/P |
| Insecta | Hymenoptera | Formicidae | *Rogeria* | sp.1 | X | | P |
| Insecta | Hymenoptera | Formicidae | *Strumigenys* | sp.1 | X | | E |
| Insecta | Isoptera | Termitidae | *Termes* | sp. | X | X | E |

| | | | | | | | |
|---|---|---|---|---|---|---|---|
| Insecta | Lepidoptera | Noctuoidea | | sp.2 | X | X | E |
| Insecta | Lepidoptera | Noctuoidea | | sp.4 | X | | E |
| Insecta | Lepidoptera | Tineoidea | | sp.3 | X | | P |
| Insecta | Orthoptera | Mogoplistidae | | jovem | 1 | | E |
| Insecta | Orthoptera | Phalangopsidae | *Paraclodes* | sp. | 1 | 6 | P |
| Insecta | Orthoptera | Phalangopsidae | *Phalangopsis* | sp. | 1 | 6 | P |
| Insecta | Psocoptera | Epipsocidae | *Mesepipsocus* | sp.1 | | X | P |
| Insecta | Psocoptera | Ptiloneuridae | *Ptiloneura* | sp.1 | | X | E |
| Amphibia | Anura | Strabomantidae | *Pristimantis* | cf. *fenestratus* | 4 | 1 | E/P |
| Mammalia | Chiroptera | Emballonuridae | *Peropteryx* | sp. | 1 | | E |
